# Identification of unmet palliative care needs of nursing home residents: A scoping review protocol

**DOI:** 10.1371/journal.pone.0306980

**Published:** 2024-08-08

**Authors:** Patrice Crowley, Mohamad M. Saab, Nicola Cornally, Isabel Ronan, Sabin Tabirca, David Murphy

**Affiliations:** 1 Catherine McAuley School of Nursing and Midwifery, University College Cork, Cork, Ireland; 2 School of Computer Science and Information Technology, University College Cork, Cork, Ireland; Xiamen University - Malaysia Campus: Xiamen University - Malaysia, MALAYSIA

## Abstract

**Introduction:**

Nursing home residents often have life limiting illnesses in combination with multiple comorbidities, cognitive deficits, and frailty. Due to these complex characteristics, a high proportion of nursing home residents require palliative care. However, many do not receive palliative care relative to this need resulting in unmet care needs. To the best of our knowledge, there have been no literature reviews to synthesise the evidence on how nursing home staff identify unmet palliative care needs and to determine what guidelines, policies, and frameworks on identifying unmet palliative care needs of nursing home residents are available.

**Aim:**

The aim of this scoping review is to map and summarise the evidence on identifying unmet palliative care needs of residents in nursing homes.

**Methods:**

This scoping review will be guided by the JBI Manual for Evidence Synthesis. The search will be conducted in CINAHL, MEDLINE, Embase, Web of Science, APA PsycINFO, and APA PsycArticles. A search of grey literature will also be conducted in databases such as CareSearch, Trip, GuidelineCentral, ClinicalTrials.gov, and the National Institute for Health and Care and Excellence website. The search strategy will be developed in conjunction with an academic librarian. Piloting of the screening process will be conducted to ensure agreement among the team on the eligibility criteria. Covidence software will be used to facilitate deduplication, screening, and blind reviewing. Four reviewers will conduct title and abstract screening. Six reviewers will conduct full text screening. Any conflicts will be resolved by a reviewer not involved in the conflict. One reviewer will conduct data extraction using pre-established data extraction tables. Results will be synthesised, and a narrative synthesis will be used to illustrate the findings of this review. Data will be presented visually using tables, figures, and word clouds, as appropriate.

## Introduction

### Background and rationale

Palliative care is a holistic approach to care that aims to improve the quality of life of those living with a life limiting illness. A key element of palliative care is the early identification of needs to facilitate care planning and early intervention [1, 2]. Palliative care needs include a person’s physical, psychological, spiritual, and social needs [1–3]. When palliative care is implemented early, it can benefit individuals through reduced crisis hospital admissions, improvement in overall quality of life, and better end of life outcomes [4–6]. Despite the recognised benefits of palliative care, the World Health Organization estimated that of the approximate 56.8 million people worldwide who require palliative care annually, only 14% receive it [2]. Therefore, 48.8 million people live with unmet palliative care needs every year.

A key setting in which palliative care needs can be addressed is nursing homes. A longitudinal study spanning over three years, conducted in the United Kingdom by Vossius et al. [7] found that approximately 33% of nursing home residents die annually. In addition, the Central Statistics Office, reported that in Ireland, 17% of total deaths in 2021 occurred in a nursing home [8]. For those over the age of 75, that figure increases to 25% of the total deaths that year occurring in a nursing home. Residents of nursing homes often have life limiting illnesses along with multiple comorbidities, cognitive deficits, and frailty which are associated with reduced quality of life and high levels of dependency [9–11].

These complex characteristics, as well as high mortality, cause nursing home residents to have a large number of care needs and are often suited to a palliative care approach. Despite this, findings from the literature demonstrate that nursing home residents do not receive palliative care relative to their needs [12–14]. Furthermore, when palliative care is provided in a nursing home, the quality has been found to be sub-optimal [15]. This lack of, or low quality, palliative care results in a high proportion of unmet palliative care needs of nursing home residents, with consequent reduced quality of life [13].

A systematic review by Carpenter et al. [16] examined interventions to improve palliative care provision in nursing homes. These included a focus on training staff, advance care planning, and establishing a palliative care team. The recommendations from this review proposed future research to focus on identifying residents with the greatest need. Similarly, Hawley, highlighted identifying unmet palliative care needs, along with lack of education of staff and limited resources, as a barrier to palliative care provision [17].

In other settings (e.g. hospital, emergency department, and primary care), screening tools have been used to identify unmet palliative care needs [18–22]. Screening tools are instruments that are widely used in healthcare to identify the presence or risk of a particular disease or problem. In the context of unmet palliative care needs, screening tools use different indicators, for example, reduced mobility, increasing dependence, and weight loss to identify those with unmet needs [23, 24].

To the best of the authors’ knowledge, only one review exists on screening tools that identify unmet palliative care needs of nursing home residents [25]. It was found that of the four tools reviewed, only one screening tool met the COnsensus-based Standards for the selection of health Measurement INstruments (COSMIN) criteria which are used for assessing the methodological quality of tools [26]. This was the Necesidades Paliativas (NECPAL) tool which was originally developed in Spain by Gómez-Batiste et al. [27]. However, Cole et al. [25] evaluated the NECPAL further, using the Grading of Recommendations, Assessment, Development, and Evaluations (GRADE) Framework. This framework is used to evaluate and rank evidence to make clinical practice recommendations [28]. Based on the GRADE framework, Cole et al. [25] determined the NECPAL as having low quality evidence indicating uncertainties with the tool’s true measurement properties. Furthermore, Cole et al. [25] outline that there was a lack of reporting on some assessments of measurement properties of the tool such as structural validity, internal consistency, and reliability. This resulted in a conclusion that there was not enough evidence to recommend the NECPAL as a ‘gold standard’ tool for use in nursing home settings. Ultimately, the recommendations of this review argued for the development of a specifically designed screening tool for use in nursing home settings to capture the unique needs of this population.

While this scoping review will also determine the available screening tools for identifying unmet palliative care needs of nursing home residents, there are distinct differences from the systematic review by Cole et al. [25]. First, this review has an additional focus on what methods (not limited to screening tools) nursing home staff use to identify unmet palliative care needs. Second, this review will outline the characteristics of the tools and map the specific indicators for identifying unmet palliative care needs. Third, this review will identify palliative care referral pathways as an outcome of the screening process. Finally, relevant guidelines, policies, and frameworks will be identified and reviewed.

### Review questions

The key concepts for the research questions for this review were developed using the population, concept, and context framework as recommended in the JBI Manual for Evidence Synthesis [29]. The three research questions are as follows:

1. What methods do staff use to identify unmet palliative care needs of residents in nursing homes?
2. What screening tools have been developed to identify unmet palliative care needs of nursing home residents?
  - What are the characteristics of the tools (including relevant indicators)?
  - How are the tools integrated into routine care and what are their assessment processes?
  - What were the development and evaluation processes of the tools?
3. What guidelines, policies, or frameworks exist regarding identifying unmet palliative care needs of nursing home residents?

## Methods

### Introduction

A scoping review looks at a topic in a broad perspective, often used to identify and map relevant literature. Scoping reviews are used to identify knowledge gaps in the research as well as to identify key concepts or characteristics and the types of studies conducted related to a broad topic. A scoping review may also be a precursor to determine the feasibility of a systematic review [29–31]. Therefore, given the broad aim and questions of this review, it was determined that a scoping review was the most appropriate approach.

This scoping review protocol was guided by the JBI Manual for Evidence Synthesis [29]. In addition, the Preferred Reporting Items for Systematic Reviews and Meta-Analyses (PRISMA) extension for protocols was used to report this review protocol [32]. The full preregistration for this scoping review can be found at https://osf.io/3pf8e.

A preliminary search was conducted in CINAHL, MEDLINE, the Cochrane Library, PROPSERO, Open Science Framework and Google Scholar. One systematic review was identified on this topic as outlined above [25]. There was no other existing reviews or registered protocols on this topic.

### Eligibility criteria

The review eligibility criteria were predetermined using the population, concept and, context framework as recommended by JBI [33]. The eligibility criteria are as follows:

#### Population

Nursing home residents.

#### Concept

The concept of interest is unmet palliative care needs which has varying or limited definitions within the literature [20, 21, 34]. Ventura et al. [35] suggest this may be due to a presumed common understanding of the individual words. Considering this as well as the aforementioned definition of palliative care needs, we define an unmet palliative care need as an unfulfilled physical, psychological, social, or spiritual need [1–3]. These associated needs must have the potential to be fulfilled through palliative care for example, with effective symptom management, initiation of advance care planning, or referral for hospice care.

Papers reporting on the development and/or testing of a screening tool for identifying unmet palliative care needs. Papers reporting on the identification of unmet palliative care needs of nursing home residents by staff. Papers reporting on characteristics of residents eligible for palliative care. Clinical guidelines, frameworks, and policies that provide guidance on best practice for identifying unmet palliative care needs.

#### Context

Nursing homes, which are defined as a facility providing long term 24-hour care to residents with varying levels of independence, needing assistance with activities of daily living [36].

Studies reporting on multiple settings and/or multiple populations will be included only where the data for nursing home residents are reported separately. Grey literature such as guidelines, theses, and legislation will be considered for any relevant information on identifying unmet palliative care needs. There will be no limit placed on the methodology (qualitative, quantitative, mixed-methods, reviews) of the included papers as during the preliminary search, papers of a variety of methodologies were deemed relevant.

#### Exclusion criteria

Articles focused on assessment of symptom management of people receiving palliative care will be excluded. Articles focused on tools used to identify acute deterioration such as Early Warning Score tools will also be excluded [37]. Articles focused on prognostic tools such as the Palliative Performance Scale will be excluded as these tools have a focus on life expectancy rather than identifying resident’s needs and supporting their quality of life [38]. Studies conducted in settings such as acute care, emergency department, primary care, and hospice will be excluded as these are not the setting of interest. There will be no limitation on country or healthcare system for this scoping review. The full eligibility criteria are detailed in Table 1.

**Table 1:** The review eligibility criteria using the population, concept, context framework [33].

Inclusion	Exclusion
**Population** • Nursing home residents	**Population** • Not a resident of a nursing home
**Concept** Unmet palliative care needs^1^: • Papers reporting the development, testing, or use of a screening tool to identify unmet palliative care needs • Papers reporting methods of identifying unmet palliative care needs • Papers reporting characteristics of residents eligible for palliative care • Clinical guidelines, frameworks, and policies on identifying unmet palliative care needs	**Concept** • Papers on assessing symptom management of people already receiving palliative care • Papers on identifying acute deterioration • Tools that focus solely on one indicator such as pain • Prognostic tools
**Context** • Nursing home setting^2^	**Context** • Any other setting e.g. acute care, primary care, emergency department, and hospice
**Types of evidence** • Papers of all research design types will be considered such as mixed-method, qualitative, quantitative, and reviews • Grey literature such as guidelines, theses, and policies	

^1^ We define an unmet palliative care need as an unfulfilled physical, psychological, social, or spiritual need [1–3]. These associated needs must have the potential to be fulfilled through palliative care for example, with effective symptom management, initiation of advance care planning, or referral for hospice care.

^2^ a facility providing long term 24-hour care to residents with varying levels of independence needing assistance with activities of daily living [36].

### Information sources and search strategy

The search strategy will be developed by PC, NC, and MMS in conjunction with an academic librarian. As per the JBI Manual for Evidence Synthesis, a three step search strategy will be used [29]. An initial search of two databases (Cumulative Index of Nursing and Allied Health Literature [CINAHL] and MEDLINE) will be conducted using synonyms of keywords. This search will be used to identify further keywords and terms used in the literature to ensure the search is optimised to find relevant papers.

The three search concepts are: palliative care, nursing homes, and screening/guidelines (see Table 2).

**Table 2:** MEDLINE search strategy.

Search	Query	Records retrieved
**#1**	**MeSH terms:** "Hospice and Palliative Care Nursing" OR "Palliative Medicine" OR "Palliative Care" OR "Terminal Care" **OR** **Title/Abstract:** palliat* OR "end of life" OR "end-of-life" OR "EOL" OR terminal OR terminally OR hospic* OR dying OR "last year of life"	**681,555**
**#2**	**MeSH terms:** "Nursing Homes" OR "Residential Facilities" OR "Assisted Living Facilities" OR "Homes for the Aged" **OR** **Title/Abstract:** "nursing home*" OR "long term care" OR "long-term care" OR "retirement home*" OR "residential care" OR "residential home*" OR "residential facility" OR "residential facilities" OR "assisted living" OR "assisted-living" OR "old-age home" OR "old-age homes" OR "Homes for the Aged" OR "care home" OR "care homes"	**94,754**
**#3**	**MeSH terms:** "Needs Assessment" OR "Nursing Assessment" OR "Practice Guideline" **OR** **Title/Abstract:** (screen* OR identif* OR assess* OR predict*) N3 (need OR needs OR tool* OR instrument OR instruments OR evaluat* OR questionnaire* OR survey* OR "check list* OR checklist* OR score OR scores OR scoring"OR scale*OR detect* OR "decision tool*" OR "decision aid*"OR "decision tree*" OR "decision support*" OR algorithm* OR indicator* OR criteria OR criterion OR guideline OR guidelines OR guide OR guides OR policy OR policies OR framework OR pathway	**1,198,651**
**#4**	#1 AND #2 AND #3	**749**

Specific subject headings for each relevant database will be determined to broaden the search. The search strategy will be customised for each of the databases. Piloting will be conducted before the full search strategy is applied to ensure agreement on eligibility criteria among the reviewers. This will start by choosing a random sample of 25 titles and abstracts. The team will screen these independently using the inclusion and exclusion criteria. Any discrepancies will be resolved through discussion among the team. Official screening will not begin until there is agreement of 75% or more.

After refinement of the search strategy and eligibility criteria, a second systematic search of the literature will be conducted in CINAHL, MEDLINE, Embase, Web of Science, APA PsycINFO, and APA PsycArticles. A search of the grey literature will also be conducted in databases such as: CareSearch, Trip, and GuidelineCentral. In addition, ClinicalTrials.gov and the National Institute for Health and Care and Excellence website will be searched. The grey literature will be searched for relevant documents such as guidelines, policies, reports, and theses/dissertations related to identifying unmet palliative care needs of nursing home residents. No limit will be applied on the year of publication or language.

The third step of the strategy is to read the reference lists of all included papers to identify further relevant sources for inclusion. The search may be modified during the process; however, full details of any minor modifications will be outlined in the final write up of the scoping review. If major modifications must be made to the search strategy, then a revised protocol will be submitted. The search will be re-run prior to final analysis to ensure the review is up to date upon publication. Zotero software (Corporation for Digital Scholarship, Virginia, United States of America, 2024) will be used for citation management.

### Study selection and screening

Records identified from the database searches will be uploaded to Covidence software (Veritas Health Innovation, Health Innovation, Melbourne, Australia, 2024) and deduplicated automatically. Covidence software will be used to facilitate screening and blind reviewing. Selection of papers for inclusion will be based on the eligibility criteria within this protocol. Title and abstract screening will be divided amongst four reviewers (PC, MMS, NC, and IR) such that every paper has been independently reviewed by two reviewers. If during the search, an article does not have an abstract (i.e. only a title) then it will automatically move forward to the full text review stage. At the full text review stage, all full texts will be retrieved and uploaded to Covidence software. The subsequent full texts will be divided and reviewed by six reviewers to determine their applicability (PC, MMS, NC, IR, ST, and DM). Again, at this stage every paper will be independently reviewed by two reviewers. Any screening conflicts at the title and abstract or full text stage will be resolved by a reviewer that is independent to the conflict. Reasons for studies being excluded will be documented and outlined in the write up of the review. A PRISMA flow diagram (see S1 Fig) by Page et al. [39] will be used to illustrate the study identification, screening, and selection process.

### Data extraction

To meet the objectives of the review it was determined that three data extraction tables were required (see S1–S3 Tables). One to extract the screening tool characteristics, one to extract data on the assessment, use, development, and implementation of the screening tools and one to extract data from the grey literature.

Data from the included articles will be extracted under the following headings: reference; country; sample size; research design; screening tool name; purpose; tool format; specificity to disease; number of items; scoring system; indicators; assessor; frequency of assessment; time needed to complete; referral pathway; phases of development; stakeholder engagement; educational intervention.

One reviewer will conduct data extraction (PC). Two reviewers will check the data extraction tables for accuracy (NC, MMS). The development of the data extraction tables was broadly based on the template in the JBI Manual for Evidence Synthesis [29]. They were later adapted by the team to meet the aim and objectives of this scoping review. The data extraction tables will be piloted, discussed, and refined among the reviewers. Any changes to the data extraction tables will be outlined in the final published scoping review. If any data are missing from a study, authors will be contacted. Any missing data will be coded as ‘not reported’ and ambiguous data will be coded as ‘unclear’.

### Synthesis and presentation of results

Results will be synthesised qualitatively. The JBI Manual for Evidence Synthesis as well as the paper by Pollock et al. [33] will guide the synthesis and presentation of results for this scoping review [29]. As advised by JBI, the presented data will take a more descriptive than analytical approach. Descriptions will be outlined such as the number of screening tools, characteristics of the tools, country of origin, sample size, and the types of documents retrieved (e.g. primary papers, reviews, policies, guidelines) as appropriate. Characteristics of the included studies will also be described. Basic qualitative content analysis will be conducted on the indicators of identifying unmet palliative care needs [33]. The frequency of each indicator will be displayed on a table or a word cloud. All methods of presenting the data such as tables and figures will be accompanied with a narrative synthesis. All of the collected data will be correlated to the aim and objectives of this scoping review with a narrative summary. The approach to data analysis and synthesis may change at any stage during the review. However, the finalised approach will be outlined in the final write-up of the scoping review. The completed scoping review will be published with open access. Therefore, all data including data extraction tables will be publicly available either within the published scoping review or provided as supplementary material.

### Ethics and dissemination

Ethical approval is not required for this scoping review. This scoping review will be submitted for publication to a peer reviewed academic journal. Findings from this review will also be disseminated at a relevant academic conference.

## Conclusion

The overarching aim of this scoping review is to map the literature on identifying unmet palliative care needs of nursing home residents. Specific methods used by nursing home staff, including screening tools, palliative care referral pathways, and relevant guidelines, policies, and frameworks will be identified. To the best of the authors’ knowledge, this will be the first review mapping the methods, guidelines, and indicators for identifying unmet palliative care needs of nursing home residents. This will advance the field by providing valuable insights into the identification of unmet palliative care needs of nursing home residents. Furthermore, this research team intends to use this information to conduct primary research in the area of identifying unmet palliative care needs of nursing home residents.

## Supporting information

S1 Checklist PRISMA-P checklist. (DOC)

S1 Fig PRISMA flow diagram. (PDF)

S1 Table Data extraction Table 1- screening tool characteristics. (DOCX)

S2 Table Data extraction Table 2- assessment, use, development, and evaluation of screening tools. (DOCX)

S3 Table Data extraction Table 3- guidelines, policies, and frameworks. (DOCX)
