# Supplementary figures and images for: Identification of unmet palliative care needs of nursing home residents: A scoping review protocol
Source: PLoS One. 2024 Aug 8;19(8):e0306980. doi: 10.1371/journal.pone.0306980 (PMC11309440; doi:10.1371/journal.pone.0306980)

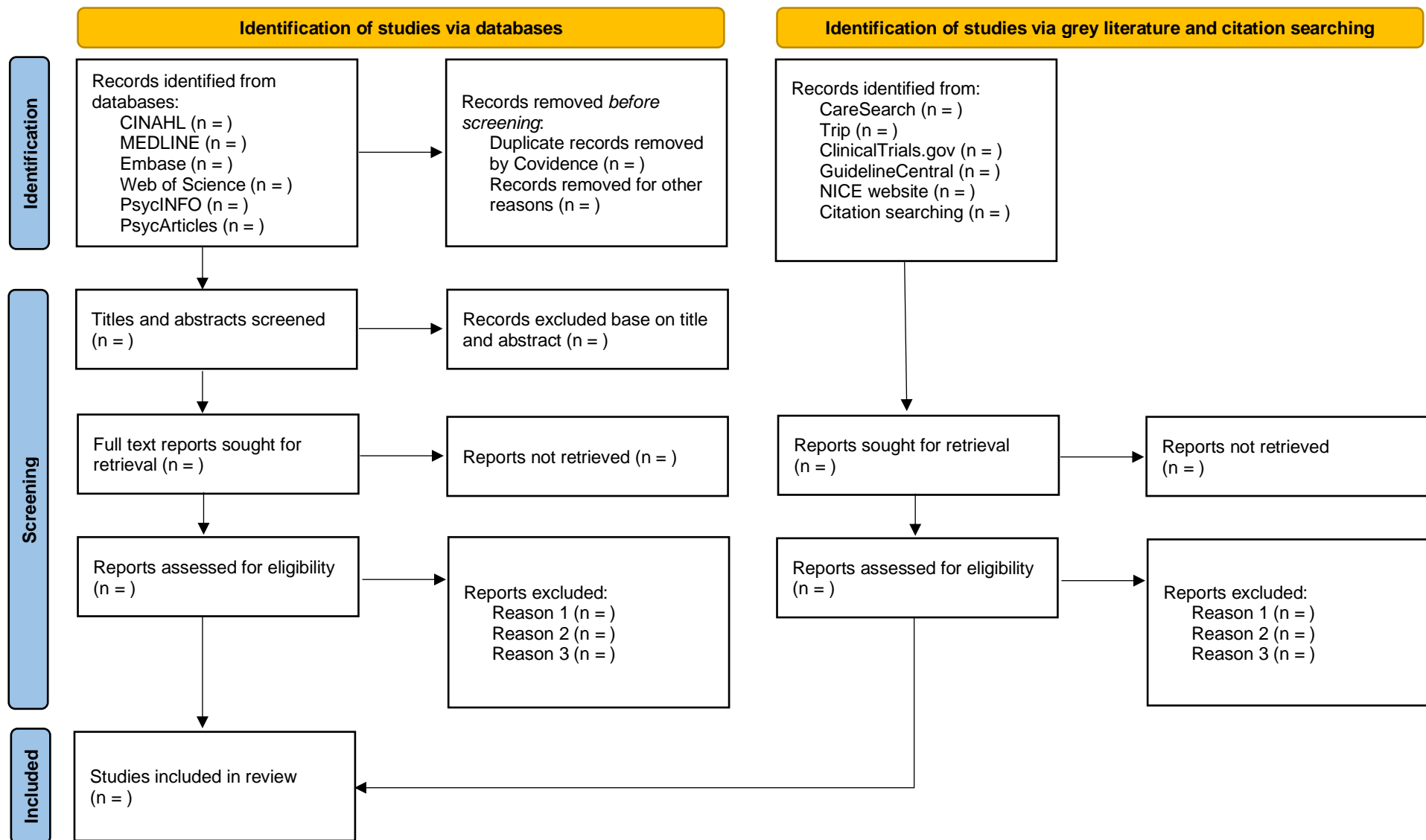

Supplement: S1 Fig — (PDF) [file pone.0306980.s002.pdf]
