# Supplementary material for: Identification of unmet palliative care needs of nursing home residents: A scoping review protocol
Source: PLoS One. 2024 Aug 8;19(8):e0306980. doi: 10.1371/journal.pone.0306980 (PMC11309440; doi:10.1371/journal.pone.0306980)
Supplement: S1 Table — (DOCX) [file pone.0306980.s003.docx]

| **Reference and country** | **Sample size and design** | **Tool name and purpose** | **Tool format and specificity to disease** | **Number of items and scoring system** | **Indicators** |
| --- | --- | --- | --- | --- | --- |
|  |  |  |  |  |  |

**S1 Table. Data extraction table 1- Screening tool characteristics**
