# Supplementary material for: Identification of unmet palliative care needs of nursing home residents: A scoping review protocol
Source: PLoS One. 2024 Aug 8;19(8):e0306980. doi: 10.1371/journal.pone.0306980 (PMC11309440; doi:10.1371/journal.pone.0306980)
Supplement: S2 Table — (DOCX) [file pone.0306980.s004.docx]

**S2 Table. Data extraction table 2-** **Assessment, use, development, and evaluation of screening tools**

|  | **Assessment and Use** | | **Development** | | | **Evaluation** |
| --- | --- | --- | --- | --- | --- | --- |
| **Tool name** | **Assessor and frequency of assessment** | **Time needed to complete and referral pathway** | **Did a guideline, policy, or framework guide the development?** | **Phases of development** | **Stakeholder engagement** | **How was the tool evaluated?** |
|  |  |  |  |  |  |  |
