# Supplementary material for: Identification of unmet palliative care needs of nursing home residents: A scoping review protocol
Source: PLoS One. 2024 Aug 8;19(8):e0306980. doi: 10.1371/journal.pone.0306980 (PMC11309440; doi:10.1371/journal.pone.0306980)
Supplement: S3 Table — (DOCX) [file pone.0306980.s005.docx]

**S3 Table. Data extraction table 3- Guidelines, policies, and frameworks**

| **Document Details** | | | **Document Characteristics** | | **Assessment and Implementation** |
| --- | --- | --- | --- | --- | --- |
| **Reference, country** | **Document name** | **Document aim** | **Type of document, intended users and specificity to disease** | **Indicators** | **Recommended frequency of assessment and referral pathway** |
|  |  |  |  |  |  |
